# *Clostridium difficile* infection health disparities by race among hospitalized adults in the United States, 2001 to 2010

**DOI:** 10.1186/s12879-016-1788-4

**Published:** 2016-08-27

**Authors:** Jacqueline R. Argamany, Andrew Delgado, Kelly R. Reveles

**Affiliations:** 1 The University of Texas College of Pharmacy, 2409 University Avenue, A1900, Austin, TX 78712 USA; 2 The University of Texas Health Science Center Pharmacotherapy and Education Research Center, 7703 Floyd Curl Drive, MC-6220, San Antonio, TX 78229 USA

**Keywords:** Race, Disparity, *Clostridium difficile*

## Abstract

**Background:**

Recognition of health disparities in *Clostridium difficile* infection (CDI) is an initial step toward improved resource utilization and patient health. The purpose of this study was to identify health disparities by black vs. white race among hospitalized adults with CDI in the United States (U.S.) over 10 years.

**Methods:**

This was a retrospective analysis of the U.S. National Hospital Discharge Surveys from 2001 to 2010. Eligible cases included adults with an ICD-9-CM code for CDI (008.45). Patients with missing race or “other race” were excluded. The primary outcome, CDI incidence, was calculated as CDI discharges per 1,000 total discharges. Data weights were used to determine national estimates. Secondary outcomes included in-hospital mortality, hospital length of stay (LOS), and severe CDI. Comparisons were made using bivariable analyses. Race was assessed as an independent risk factor for CDI outcomes using logistic regression or proportional hazards models.

**Results:**

These data represent 1.7 million CDI discharges, where 90 % of patients were identified as white and 10 % black. Blacks differed from whites with respect to all baseline characteristics (*p <*0.0001). CDI incidence was significantly higher in whites compared to blacks (7.7/1,000 discharges vs. 4.9/1,000 discharges, *p <* 0.0001). Blacks had higher mortality (7.4 % vs. 7.2 %, *p <* 0.0001), LOS >7 days (57 % vs. 52 %, *p <* 0.0001), and severe CDI (24 % vs. 19 %, *p <* 0.0001). In multivariable analyses, black race was a positive predictor of mortality (OR 1.12, 95 % CI 1.09–1.15) and severe CDI (OR 1.09, 95 % CI 1.07–1.11), and negative predictor for hospital LOS (OR 0.93, 95 % CI 0.93–0.94).

**Conclusions:**

CDI incidence was higher for white patients; however, black race was independently associated with mortality and severe CDI.

## Background

Efforts are being made to eliminate health disparities in the United States (U.S.); however, disparities due to race, ethnicity, geographics, and socioeconomics continue to exist [1–4]. Prior studies have reported reduced access to primary healthcare services among minority patients, potentiating the likelihood of suboptimal patient outcomes [5–8]. Infectious diseases are the second-leading contributor to racial disparities in healthcare after cardiovascular disease [9]. Health disparities have been specifically reported in infectious diseases, though studies are limited. Compared to white patients, black patients have an increased incidence and severity of sepsis and receive a lower quality of care for severe infections [10–12]. Few studies have examined the impact of disparities on other infections, such as *Clostridium difficile* infection (CDI) [3, 13].

CDI is a common infection with increasing incidence in U.S. hospitals as well as the community [14, 15]. Symptoms of infection range from mild, uncomplicated diarrhea to more severe manifestations with complications including sepsis, renal failure, ileus, toxic megacolon, perforated intestine, or death [16–18]. The incidence of and outcomes from CDI might differ by race due to factors that influence the gastrointestinal microbiome, such as medications and diet, and socioeconomic factors, including insurance status and access to care. While research suggests black patients are less likely to receive broad-spectrum antibiotics, they are more likely to have a longer admission to a hospital emergency department and have higher rates of hospital readmission, which could impact patient health outcomes [19–22]. Despite these associations, few studies have evaluated racial health disparities in CDI.

Recognition of health disparities in CDI is an initial step towards more targeted resource utilization and improved patient health. The goal of this study was to identify health disparities by black vs. white race in CDI incidence and health outcomes among hospitalized adults with CDI in the U.S. over a 10-year period.

## Methods

### Data source

This study utilized data from the Centers for Disease Control and Prevention’s National Hospital Discharge Survey (NHDS). The NHDS is a national probability sample of non-federal, short-stay hospital discharges in the U.S. A complex, three-stage sampling methodology allows the user to apply data weights to derive national estimates representative of the U.S. population [23].

The survey data include patient demographics, such as age, gender, self-reported race, and marital status, as well as year of discharge, payment sources, geographic region, hospital length of stay (LOS), and hospital discharge status. Diagnoses and procedures are also reported as *International Classification of Diseases, 9th Revision, Clinical Modification* (ICD-9-CM) codes. NHDS data have previously been used in several infectious diseases epidemiological studies, including healthcare-associated infections [14, 24, 25].

### Study design

This was a retrospective analysis of all patients discharged from U.S. hospitals from 2001 to 2010. Eligible cases included adults at least 18 years of age with a principal or secondary ICD-9-CM discharge diagnosis code for CDI (008.45). Patients with missing race or “other race” were excluded.

Patient baseline characteristics were classified based on the categories provided in the NHDS for patient sex, hospital size (6–99 beds, 100–199 beds, 200–299 beds, 300–499 beds, or ≥500 beds), hospital ownership (proprietary, government, or nonprofit), and admission type (emergency, urgent, or elective). Other patient characteristics were classified by limited definitions designed to encompass NHDS categories: race (white, black, and other), expected primary source of payment (private, Medicare, Medicaid, self-pay, and other), and admission source (emergency room, transfer, referral, and other).

Health outcomes in this study included in-hospital mortality, hospital LOS, and any severe CDI. The “Discharge Status” item of the NHDS was used to determine patient mortality. This represents all-cause, in-hospital mortality for patients with CDI. Hospital LOS was extracted from the “Days of Care” item of the NHDS and was presented as medians (interquartile ranges). In this study, severe CDI was indicated by cases with a principal or secondary ICD-9-CM code for at least one of the following: septicemia (038.x), septic shock (785.52), acute renal failure (584.x), toxic megacolon (558.2), prolonged ileus (560.1), perforated intestine (569.83), or colectomy (45.7x).

### Statistical analyses

First, baseline patient demographics were summarized using medians (interquartile ranges) for continuous variables and counts (percentages) for categorical variables. All baseline characteristics were assessed for multicollinearity using the Spearman rank correlation. Correlation coefficients were then converted to variance inflation factors (VIF) using the following equation: VIF = 1/(1-R^2^). Two variables were considered highly correlated if they had a VIF >10 and were statistically significant at an alpha <0.0001. We compared baseline characteristics between races using bivariable analyses calculated using the chi-square test for categorical variables and Wilcoxon rank-sum test for continuous variables. Next, we determined the overall CDI incidence rate using CDI discharges as the numerator, as identified in our cohort, and total discharges as the denominator. Total discharges were derived from the composite NHDS data, which include all CDI and non-CDI patients. Incidence by race was calculated as CDI discharges per race category divided by total discharges per race category. Data weights were applied to derive national estimates and incidence rates were presented as CDI discharges per 1,000 total discharges.

CDI incidence, patient mortality, hospital LOS, and severe CDI were characterized overall and by race. CDI incidence was compared by race using the z-test. Health outcomes were compared using the chi-square or Wilcoxon rank sum test. Risk for CDI health outcomes was analyzed using multivariable logistic regression to calculate adjusted odds ratios (aOR) and 95 % confidence intervals (CI) for mortality and any severe CDI using white race as the reference category. A Cox proportional hazards model was used to calculate the aOR and 95 % CI for hospital LOS, which allowed us to censor those CDI patients who died in the hospital. The regression models controlled for patient-specific variables, including age ≥65 years, sex, geographic region, hospital size, hospital ownership, other infectious diseases as indicated by ICD-9-CM codes (skin infection, pneumonia, bacteremia, device-related infections, urinary tract infection, and endocarditis), principal or secondary CDI diagnosis, other common principal diagnoses (e.g., cancer, congestive heart failure, chronic obstructive pulmonary disease [COPD], myocardial infarction, diabetes, cerebrovascular disease, and human immunodeficiency disease [HIV]), principal payment source, admission type, and admission source. The mortality and hospital LOS models also included severe CDI as a covariate. Statistical significance was indicated by *p <* 0.0001 due to the large sample size provided by the NHDS. JMP 10.0® (SAS Corp, Cary, NC) was used for all statistical comparisons.

## Results

### Baseline characteristics

The patient baseline characteristics are provided in Table 1. Overall, these data represent approximately 1.7 million CDI discharges from U.S. hospitals over the study period. Of these patients, 90 % were white and 10 % were black. Approximately 500,000 patients were excluded because of a missing race code or classification as “other race.” Overall, patients were predominately female (59 %) and at least 65 years old (71 %). Black and white patients with CDI significantly differed with respect to patient age, sex, geographic region, hospital size and ownership, principal payment source, admission type and source, and principal diagnoses. White patients were a median (interquartile range) 76 (63–83) years of age, while black patients were much younger [65 (50–78) years]. Black patients were more likely than white patients to be discharged from a hospital with over 500 beds (25 % and 11 %, respectively), and were more likely to have Medicaid as their principal payment source (17 % and 5 %, respectively). Additionally, black patients were also more likely to have an emergent admission type (78 % and 65 %, respectively). No baseline characteristics had a VIF >2, indicating low multicollinearity among variables.

**Table 1:** Baseline characteristics

Demographic	Overall (*n =* 1,676,903)	White (*n =* 1,503,189)	Black (*n =* 173,714)	*P-*Value^a^
Age (years), median (IQR)	75 (61–83)	76 (63–83)	65 (50–78)	<0.0001
Age ≥65 years, %	71	73	50	<0.0001
Female sex, %	59	59	58	<0.0001
Geographic region, %				<0.0001
Northeast	32	33	28	
Midwest	18	18	15	
South	37	35	49	
West	13	14	8	
Hospital size, %				<0.0001
6–99 beds	20	21	12	
100–199 beds	23	24	11	
200–299 beds	23	23	21	
300–499 beds	21	21	31	
500+ beds	13	11	25	
Hospital ownership, %				<0.0001
Proprietary	14	15	11	
Government	10	9	15	
Nonprofit	76	76	74	
Principal payment source, %				<0.0001
Medicare	69	71	58	
Medicaid	6	5	17	
Private	21	21	19	
Self-pay	2	1	3	
Other	2	2	3	
Admission type, %				<0.0001
Emergency	67	65	78	
Urgent	20	21	16	
Elective	13	14	6	
Admission source, %				<0.0001
Emergency room	59	59	67	
Transfer	16	16	13	
Referral	17	17	14	
Other	8	8	6	
Selected principal diagnoses, %				
*Clostridium difficile* infection	32	32	31	<0.0001
Pneumonia	4	5	3	<0.0001
Urinary tract infection	3	2	5	<0.0001
Cancer	3	3	3	<0.0001
Congestive heart failure	2	2	2	<0.0001
COPD	2	2	1	<0.0001
Myocardial infarction	1	1	1	0.0294
Diabetes	1	1	2	<0.0001
Cerebrovascular disease	1	1	2	<0.0001
HIV	<1	<1	2	<0.0001

*IQR* interquartile range, *COPD* chronic obstructive pulmonary disease, *HIV* human inmmunodeficiency virus

^a^ *P-*values reflect comparisons between white and black patients

### CDI incidence

**Fig. 1:**
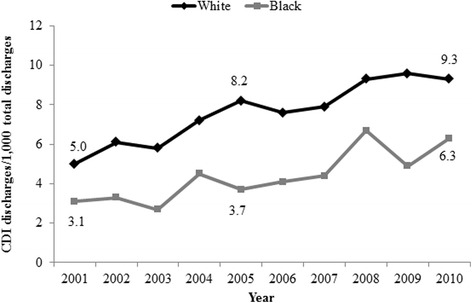
*Clostridium difficile* infection (CDI) incidence per year for white and black patients, 2001–2010

The overall CDI incidence was 7.3 CDI discharges per 1,000 total discharges. CDI incidence was significantly higher for white patients (7.7 CDI discharges per 1,000 white discharges) as compared to black patients (4.9 CDI discharges per 1,000 black discharges) (*p <* 0.0001). From 2001 to 2010, the incidence of CDI in black and white patients increased (Fig. 1). The incidence was highest in 2009 for white patients (9.6 CDI discharges per 1,000 white discharges), and highest in 2008 for black patients (6.7 CDI discharges per 1,000 black discharges).

### Health outcomes

Health outcomes are depicted in Table 2. The median (interquartile range) hospital LOS for all hospitalized patients with CDI was 8 (5–14) days and all-cause, in-hospital mortality was 7.3 %. Hospital LOS and mortality were significantly higher for black patients (9 days and 7.4 %, respectively) as compared to white patients (8 days and 7.2 %, respectively) (*p <* 0.0001 for each comparison).

**Table 2:** Health outcomes

Outcome	Overall (*n =* 1,676,903)	White (*n =* 1,503,189)	Black (*n =* 173,714)	*P-*Value^a^
CDI incidence	7.3	7.7	4.9	<0.0001
Mortality, %	7.3	7.2	7.4	<0.0001
Hospital LOS, median (IQR)	8 (5–14)	8 (4–14)	9 (5–16)	<0.0001
Hospital LOS >7 days, %	52	52	57	<0.0001
Any severe CDI,^b^ %	20	19	24	<0.0001
Sepsis	13	12	16	
Renal failure	16	15	21	

*CDI Clostridium difficile* infection, *IQR* interquartile range, *LOS* length of stay

^a^ *P-*values reflect comparisons between white and black patients

^b^Percentages not shown for severe CDI outcomes present in <5 % of the population (shock, megacolon, ileus, perforated intestine, and colectomy)

Black race was an independent risk factor for all health outcomes. Compared to white patients, black patients had significantly greater mortality (aOR 1.12, 95 % CI 1.09–1.15, *p <* 0.0001) and were at significantly greater risk for severe CDI (aOR 1.09, 95 % CI 1.07–1.11, *p <* 0.0001). Other significant predictors of mortality included: age ≥ 65 years (aOR 2.67, 95 % CI 2.60–2.75), female sex (aOR 1.08, 95 % CI 1.06–1.10), urinary tract infection (aOR 1.36, 95 % CI 1.35–1.37), sepsis (aOR 3.54, 95 % CI 3.47–3.60), and any severe CDI (aOR 2.12, 95 % CI 2.08–2.16). Other significant predictors of severe CDI included: age ≥ 65 years (aOR 1.39, 95 % CI 1.37–1.41), private hospital admission (aOR 1.61, 95 % CI 1.57–1.65), urinary tract infection (aOR 1.36, 95 % CI 1.35–1.37), and emergency admission (aOR 1.53, 95 % CI 1.51–1.55). After censoring for inpatient mortality, black patients had a significantly shorter hospital LOS compared to white patients (aOR 0.94, 95 % CI 0.93–0.94, *p <* 0.0001).

## Discussion

In this retrospective cohort study of approximately 1.7 million hospital discharges, we identified disparities in CDI incidence and health outcomes by race. Remarkably, CDI incidence among white patients was 57 % greater than that of black patients. As CDI rates continue to increase and gaps in patient access to care continue to narrow, this disparity may change. Importantly, CDI mortality and severity were significantly worse for black patients. To our knowledge, this is the first study to find that black race is associated with poorer health outcomes in CDI. Because of the small absolute differences in mortality and severity rates between white and black CDI patients, the clinical significance of our findings is unknown, but is worth addressing in future studies due to the high cost burden associated with CDI [15].

Knowledge of healthcare disparities can be used to target resource utilization to improve patient health outcomes and eliminate disparities in the future [4]. Disparities attributed to race have been reported with respect to primary care access, as well as in surgery, sepsis, and pneumonia [5–8, 10–12, 26]. However, little research has focused on disparities associated with healthcare-associated infections [13]. Our study adds to the few prior studies examining the occurrence and recurrence of CDI where race was assessed as an independent risk factor [13, 27, 28]. In 2012, Murphy et al. published the first study to note that race and ethnicity were predictive of CDI [27]. One year later, Freedberg et al. incidentally found a higher risk of recurrent CDI in black patients in a study designed to assess the risk of CDI recurrence associated with proton pump inhibitors [28]. More recently, Bakullari et al. demonstrated significant racial and ethnic disparities in the rate of occurrence of healthcare-associated infections [13]. While the study did not find a statistically significant difference in the rate of CDI between white and black patients, it examined a much smaller population using the Medicare Patient Safety Monitoring System.

The reason for such disparities in incidence and outcomes in CDI is likely multifactorial. Among patients with CDI, black patients significantly differed from white patients with respect to all patient demographics. For example, black patients with CDI were younger than white patients and had greater use of Medicaid as a principal payment source. Considering older age is a known risk factor for CDI, the younger age of black CDI patients could have contributed to the lower CDI incidence seen for blacks in this study [15, 17]. Older age is associated with more comorbidities and health care exposures, which could increase for the risk for CDI as well as increase the likelihood for early diagnosis. The significant differences in principal payment source in our study are also likely a reflection of the younger age of the black population with CDI, as compared to the typical elderly CDI population, which relied heavily on Medicare. While this study was unable to assess for relationships between all co-morbid conditions and racial differences in CDI, the chronic, co-morbid conditions disparately affecting blacks may also increase the risk for CDI and predispose this population to overall poorer health outcomes [10]. Furthermore, disparity research suggests the most effective resource use would aim at eliminating the top racial disparities, including hypertension, diabetes, and HIV [9, 10].

Factors such as health insurance, access to care, quality of care, or health-related behavior may also contribute to this disparity [9]. According to the U.S. Census Bureau in 2012, 40.6 % of blacks relied on Medicaid or other publicly-funded insurance compared to 29.3 % of non-Hispanic whites [29]. Additionally, 17.2 % of blacks were uninsured compared to 10.4 % of non-Hispanic whites [29]. Furthermore, black patients are more likely to seek care at higher-volume hospitals than white patients and often receive care at hospitals providing a lower quality of care [12, 26, 30]. According to the Agency for Healthcare Research and Quality (AHRQ) in 2010, black patients had worse access to care than white patients for one-third of core measures and were less likely to have a specific source of ongoing care (84.7 % and 86.3 %, respectively) [31]. Decreased access to care could explain the significantly increased rate of hospital admissions via emergency department for the black population seen in our study.

Differences in medication use might also contribute to CDI disparities. White patients are more likely to receive prescription antibiotics, broad-spectrum antibiotics, and proton pump inhibitors, putting them at greater risk for disruption of the normal gastrointestinal flora, likely contributing to differential CDI incidence [19, 20, 28]. Surveys have also shown that white patients are more accepting of new prescription drugs than blacks [32].

Considering race is more relevant as a social construct than a biological construct in the United States, it is unlikely the disparities noted here are due to genetic differences in the population; however, this cannot be ruled out [33–35]. A previous study by Esper et al. suggested a genetic polymorphism in black patients in the human Toll-like receptor 2 (TLR2) could directly create a race-specific alteration in host response to Gram-positive pathogens [10]. Distribution differences in the polymorphic functional allele were previously found between African Americans and Caucasians, and given differential TLR2 promoter activity in response to interferon-gamma, contrastive pathogenesis of Gram-positive infectious organisms is possible between races [36]. Considering *Clostridium difficile* is a Gram-positive bacterium, this may warrant further research.

This study has potential limitations. First, this study relied on administrative data using ICD-9-CM coding to identify CDI and other diagnoses; therefore, it may not fully capture all CDI cases. A prior study evaluating the use of the ICD-9-CM code to identify CDI found high sensitivity (78 %) and specificity (99.7 %) compared to microbiological data [37]. This study design also precluded us from assessing other markers of disease severity, such as the McCabe or Charlson Comorbidity scores. Additionally, race was self-reported and may not accurately represent the patient population. The exclusion of patients with missing race or “other race” could limit the generalizability of the results. The NHDS excludes federal hospitals and long-term care hospitals, potentially limiting the application of our results to those patient populations. Data regarding patient ethnicity, socioeconomic status, treatment(s), prior medication(s), care access, and other patient demographics were unavailable for this study. The lack of control for these factors may have influenced results. Future studies evaluating CDI disparities should consider health insurance status or funding source, education level, medication and disease history, as well as health-related behavior issues in order to evaluate the broader scope of the problem.

## Conclusions

Despite the higher incidence of CDI among white patients, black patients had significantly higher mortality and risk for severe CDI compared to white patients. Further studies are needed to provide insights into the basis for this observation. Knowledge of existing health disparities can be used to better direct resources and improve patient care until such disparities can be eliminated or prevented.

## Abbreviations

AHRQ: Agency for healthcare research and quality
aOR: Adjusted odds ratio
CDI: *Clostridium difficile* infection
CI: Confidence interval
COPD: Chronic obstructive pulmonary disease
HIV: Human immunodeficiency virus
ICD-9-CM: International classification of diseases, 9th rev, clinical modification
LOS: Length of stay
NHDS: National hospital discharge survey
TLR2: Toll-like receptor 2
US: United States
VIF: Variance inflation factor
